# Preparation and Characterization of a Hybrid Complex of Cyclodextrin-Based Metal—Organic Frameworks-1 and Ascorbic Acid Derivatives

**DOI:** 10.3390/ma14237309

**Published:** 2021-11-29

**Authors:** Ayumi Nanri, Masaaki Yoshida, Yoshiyuki Ishida, Daisuke Nakata, Keiji Terao, Florencio Jr Arce, Gerard Lee See, Takashi Tanikawa, Yutaka Inoue

**Affiliations:** 1 Laboratory of Drug Safety Management, Faculty of Pharmacy and Pharmaceutical Sciences, Josai University, 1-1 Keyakidai, Sakado, Saitama 3500295, Japan; nanri.1202@gmail.com (A.N.); yy16310@josai.ac.jp (M.Y.); 2 CycloChem Bio Co., Ltd., 7-4-5, Minatojima-minamimachi, Chuo-ku, Kobe 6500047, Japan; yoshiyuki.ishida@cyclochem.com (Y.I.); daisuke.nakata@cyclochem.com (D.N.); keiji.terao@cyclochem.com (K.T.); 3 Laboratory of Nutri-Pharmacotherapeutics Management, Faculty of Pharmacy and Pharmaceutical Sciences, Josai University, 1-1 Keyakidai, Sakado, Saitama 3500295, Japan; tanikawa@josai.ac.jp; 4 Department of Pharmacy, School of Health Care Professions, University of San Carlos, Cebu 6000, Philippines; fvarce@usc.edu.ph (F.J.A.); glsee@usc.edu.ph (G.L.S.); 5 Pharmaceutical Sciences Division, National Research Council of the Philippines, Taguig City 1631, Philippines

**Keywords:** MOF-1, cyclodextrin, ascorbic acid, drug carrier, inclusion complex

## Abstract

Cyclodextrin-based metal–organic frameworks-1 (CD-MOF-1) prepared using potassium hydroxide, ethanol, and γ-cyclodextrin (γ-CD) has been reported as a new type of MOF for the development of pharmaceutical formulations. The present study aimed to investigate the physicochemical properties of ascorbic acid derivatives (L-ascorbyl 6-palmitate (ASCP); L-ascorbyl 2,6-palmitate (ASCDP)) complexed with CD-MOF-1 by a solvent evaporation method. Powder X-ray diffraction revealed that the crystal diffraction pattern of CD-MOF-1 changed from α-type to β-type when prepared by a solvent evaporation method. For ASCP/CD-MOF-1 = 1/2 and ASCDP/CD-MOF-1 = 1/4 evaporated samples, the crystal diffraction peaks derived from ASCP and ASCDP disappeared, indicating a β-like behavior. Differential scanning calorimetry results revealed that the endothermic peaks of evaporated samples (ASCP/CD-MOF-1 = 1/2 and ASCDP/CD-MOF-1 = 1/4) were not detected due to melting. Furthermore, intermolecular interactions were observed in the hydrogen bonds between the CH groups of the side chains of ASCP and ASCDP and the OH group of CD-MOF-1 in (ASCP/CD-MOF-1 = 1/2) and EVP (ASCDP/CD-MOF-1 = 1/4), based on the near-infrared absorption spectroscopy analysis. CD-MOF-1 did not form inclusion complexes with the lactone rings of ASCP and ASCDP, but with the lipophilic side chains. These results suggested that CD-MOF-1 may be useful in preparing novel drug carriers for ASCP and ASCDP.

## 1. Introduction

**Figure 1:**
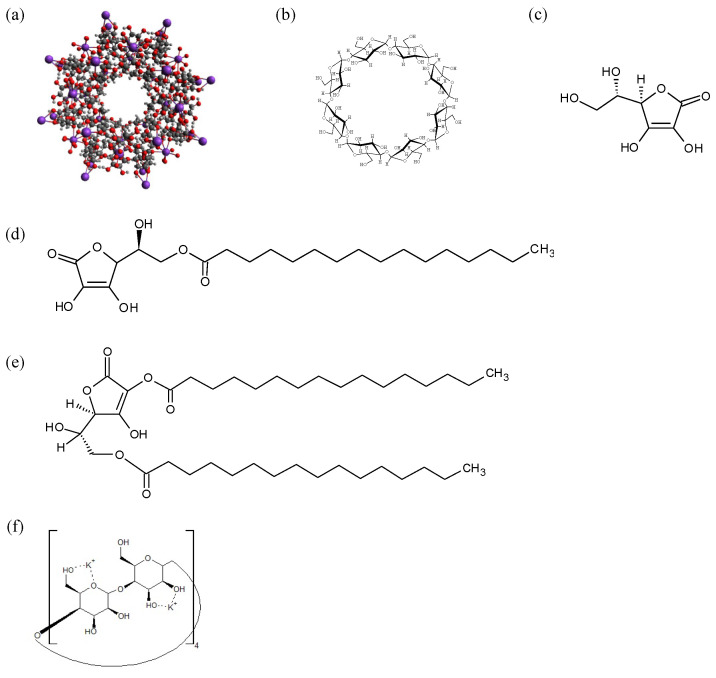
Chemical structures of: (**a**) CD-MOF-1; (**b**) γ-CD; (**c**) ASC; (**d**) ASCP; (**e**) ASCDP; (**f**) γ-CD(KOH)_2_.

Metal–organic frameworks (MOFs), in which organic ligands form clusters with metals, are expected to find a variety of applications in gas storage [1,2,3], separation technology [4,5], and sensors and catalysts [6]. However, depending on the metal selected for the synthesis of the MOFs, the toxicity of the MOFs may limit their usefulness and hinder the development and application for pharmaceutical formulations [7]. Recently, cyclodextrin-based metal–organic frameworks-1 (CD-MOF-1) prepared using potassium hydroxide, ethanol, and γ-cyclodextrin ((γ-CD, a cyclic oligosaccharide consisting of 8 units of glucose formed by α-1, 4 glycosidic linkages) has been reported as a new edible carrier MOF to address these problems [8]. Compared to activated carbon and zeolites, CD-MOF-1 has a higher surface area and shows a higher absorptivity for CO₂. Therefore, CD-MOF-1 is also used to separate a variety of organic compounds, including saturated and unsaturated aromatics, alicyclic compounds, chiral compounds, and haloaromatic compounds [9]. CD-MOF-1 has a three-dimensional microporous structure, with potassium linked to the hydroxyl groups of six γ-CD molecules (Figure 1a,b). Patyk-Kaźmierczak et al. reported that CD-MOF-1 exists in α- and β-types, depending on the conditions of the adherent water [10]. CD-MOFs have been reported to have cavity diameters of α window (7.8 Å) and β window (4.2 Å) (10). MOFs (βCD-CsOH) prepared with different cyclic oligosaccharides have a high delivery capacity for anticancer drugs such as methotrexate and 5-fluorouracil, and in vitro cytotoxicity studies using HepG2 cells have reported that these drugs show a tendency to inhibit cytotoxicity when combined with MOFs [11]. These findings suggest that CD-MOF-1 can function as a novel drug carrier, distinct from the γ-CD form.

Daily human activities often allow repeated exposure to UV light, which induces DNA damage and the production of reactive oxygen species (ROS), leading to skin pigmentation, skin aging, and skin cancer [12,13]. ROS induce collagen oxidation and promote the production of black melanin (eumelanin), which is the cause of skin blemishes and pigmentation [14]. Melanin is produced by the oxidation of tyrosine, catalyzed by the oxidase tyrosinase. Therefore, ROS-induced pigmentation is both a health and cosmetic concern. L(+)-ascorbic acid (ASC), a water-soluble vitamin with a lactone ring structure, is a potent antioxidant (Figure 1c). ASC is widely used in pharmaceutical, cosmetic, and health food products, as it scavenges ROS in the body and prevents age spots and wrinkles [15,16,17,18,19]. However, as ASC is water-soluble, it is difficult to dissolve in oil-based substrates and is susceptible to decomposition in aqueous solution by heat, oxidation, light, and heavy metals, so its use as an additive requires caution [20,21]. Recently, various ascorbic acid derivatives with improved stability against heat and oxidative degradation have attracted more attention [22,23]. L-ascorbyl 6-palmitate (ASCP) and L-ascorbyl 2, 6-palmitate (ASCDP) are derivatives of ascorbic acid combined with palmitic acid (saturated fatty acid) (Figure 1d,e). ASCP and ASCDP behave as surfactants and are potential candidates for micellization and solubilization of drugs [24,25]. ASCP and ASCDP have been used in cosmetics and as additives due to their high stability against heat and light, and because they maintain the antioxidant and melanogenesis-inhibiting properties of ASC [15]. Nevertheless, ASCP and ASCDP are lipophilic substances and are insoluble in aqueous bases, which can be an obstacle in product development.

In our previous work, we prepared inclusion complexes of Coenzyme Q10 (CoQ10) and CD-MOF-1 and evaluated their physical properties, solubility, and stability [26]. The results showed that the inclusion of the isoprene side chain of CoQ10 in the α window of CD-MOF-1 resulted in the successful formation of the complex. CD-MOF-1 was successfully solubilized in distilled water and fasted state simulated intestinal fluid (FaSSIF), and its stability was improved even under harsh conditions (temperature of 40 °C and relative humidity of 82% in the presence of saturated potassium chloride solution). Clarification of the form of inclusion with CD-MOF-1 may facilitate future pharmaceutical product development by improving the solubility, absorption, and stability of various liposoluble compounds with side chains. In this study, we focused on the ascorbic acid derivatives ASCP and ASCDP. As an initial step, we evaluated the physical properties of ASCP to determine whether it allowed inclusion of side chains in the same manner as CoQ10. Secondly, we evaluated the selectivity of the inclusion of CD-MOF-1 in compounds with multiple side chains such as ASCDP (ASCDP compared to ASCP).

## 2. Materials and Methods

### 2.1. Materials

The γ-cyclodextrin (γ-CD; Lot 801005) was provided by Cyclochem Co., Ltd. (Kobe, Japan) and stored at 40 °C and 82% RH for 7 days (Figure 1). The humidity was controlled to obtain γ-cyclodextrin 12-hydrate. Ascorbic acid palmitate (ASCP; Lot LZ2LL-EG) and ascorbic acid dipalmitate (ASCDP; Lot ISPKK-LH) were purchased from Tokyo Kasei Kogyo Co., Ltd. (Fukaya, Japan). The other reagents were purchased from Fujifilm Wako Pure Chemicals Co., Ltd. (Osaka, Japan).

### 2.2. Preparation of CD-MOF-1

CD-MOF-1 was prepared by the method reported by Samldone et al. [8]. Briefly, γ-CD (1.30 g, 1 mmol) and KOH (0.45 g, 8 mmol) were dissolved in distilled water (20 mL) in a test tube. The resulting aqueous solution was filtered, and ethanol (50 mL) was added in a conical flask and allowed to vapor-diffuse for 1 week. The colorless cubic crystals obtained were filtered and washed with ethanol and then air-dried at room temperature. CD-MOF-1 was stored at room temperature while drying in a desiccator to keep the moisture content constant. One molecule of CD-MOF-1 was designated as γ-CD(KOH)₂ and expressed as a compositional formula (Figure 1f).

### 2.3. Preparation of ASCP or ASCDP Physical Mixture and Evaporated Samples

The physical mixture (PM) was prepared by weighing ASCP and CD-MOF-1 in a molar ratio (1/1) and ASCDP and CD-MOF-1 in a molar ratio (1/1). The molar ratio was calculated using a molecular weight of 1409 g/mol to make one molecule of CD-MOF-1 as γ-CD(KOH)_2_. The weighed samples were then mixed using a vortex mixer for 1 min. For the evaporated sample (EVP), ASCP or ASCDP was dissolved in 50 mL of ethanol and mixed with CD-MOF-1 plus 100 µL of distilled water. The mixing molar ratios were ASCP/CD-MOF-1 = 1/1, 1/2 and ASCDP/CD-MOF-1 = 1/1, 1/2, 1/4. The samples were prepared by solvent removal (Rotavapor R-215, Büchi, Switzerland) at 47 °C and 58 mbar. The sample was vacuum-dried in a desiccator at room temperature. 

### 2.4. Powder X-ray Diffraction (PXRD) Measurement

A powder X-ray diffractometer (MiniFlex II, Rigaku, Tokyo, Japan) was used to check the changes in the crystalline state of the samples. Powder samples were held between glass plates to yield a flat sample plane when measurements were performed. Diffraction intensity was measured using a NaI scintillation counter. PXRD was performed using Cu Kα radiation (30 kV, 15 mA), a scan rate of 4°/min, and a scan range of 2θ = 5–40°.

### 2.5. Differential Scanning Calorimetry (DSC) Measurement

The thermal behavior of samples was investigated using a differential scanning calorimeter (Thermo plus EVO, Rigaku Co, Tokyo, Japan). All samples were weighed (approximate 2 mg) and heated and scanned at a rate of 10.0 °C/min under nitrogen gas flow (60 mL/min). Aluminum crimp pans were used for all samples.

### 2.6. Near-Infrared Absorption Spectroscopy (NIR) Measurement

Fourier transform near-infrared absorption spectroscopy (Buchi NIR Flex N-500: Nihon Buchi, Tokyo, Japan) was used to perform NIR. Measurement conditions were as follows: wave number at 10,000–4000 cm^−1^, scanning time at 8 s, and scanning temperature at 40 °C. Each sample was placed into a sample cup, and measurements were performed at intervals of 1 nm on the optical path.

### 2.7. Scanning Electron Microscopy (SEM) Measurement

SEM images were carried out using an S3000N scanning electron microscope (Hitachi High-Technologies Corporation, Tokyo, Japan) at an accelerating voltage of 15 kV. Gold was deposited on each sample for 70 s.

### 2.8. ^1^H-^1^H Nuclear Overhauser Effect Spectroscopy (NOESY) NMR Spectra Measurement 

A Varian NMR System 700 Mhz (Agilent Technologies, Santa Clara, CA, USA) was used to perform NMR. Spectroscopy was measured using an NMR spectrometer (Varian NMR System 700NB, Agilent) with an HCN probe and D_2_O. The resonance frequency was 699.6 MHz, the pulse width was 45°, the relaxation delay was 1.500 s, the temperature was 25 °C, and there were 256 increments. 

## 3. Results and Discussion

### 3.1. Evaluation of the Crystalline State

CD-MOFs are composed of γ-CDs, which possess enough cavities in their structure to contain molecules with OH groups of the γ-CDs located outside the cavity, making the interior hydrophobic. In CD-MOF-1, the OH group at the C6 position on the first face of the six γ-CDs binds to the K^+^ ion to form (γ-CD)_6_ units. This formation is further enhanced when the OH groups at the C2 and C3 positions of the neighboring γ-CD molecules bind to K^+^ ions, linking the (γ-CD)_6_ units together, and the (γ-CD)_6_ units as a whole form a body-centered cubic structure. Thus, the novel metal–organic framework (CD-MOF) consisting of γ-CD and KOH was formed by the ethanol diffusion method using alkali metal ion (i.e., K^+^) as a linker, and crystallization proceeded in aqueous solution at room temperature and pressure to obtain a porous crystal with a body-centered cubic structure.

**Figure 2:**
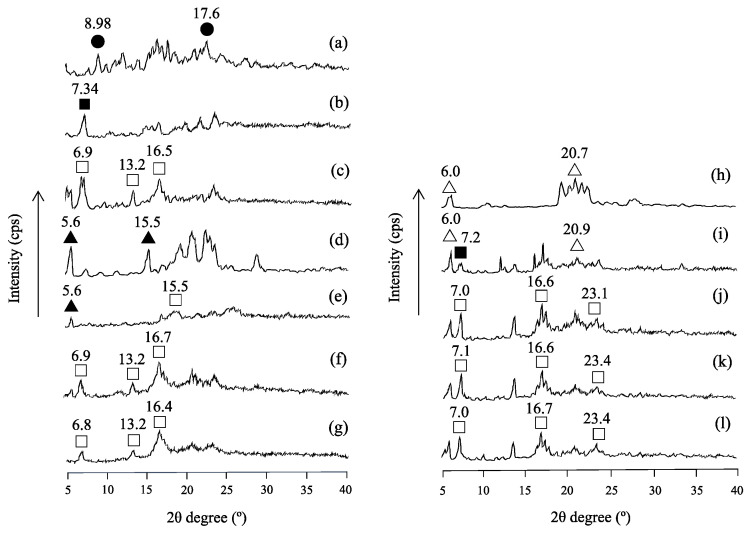
PXRD patterns of: (**a**) γ-CD; (**b**) CD-MOF-1; (**c**) EVP (CD-MOF-1); (**d**) ASCP intact; (**e**) PM (ASCP/CD-MOF-1 = 1/1); (**f**) EVP (ASCP/CD-MOF-1 = 1/1); (**g**) EVP (ASCP/CD-MOF-1 = 1/2); (**h**) ASCDP intact; (**i**) PM (ASDCP/CD-MOF-1 = 1/1); (**j**) EVP (ASCDP/CD-MOF-1 = 1/1); (**k**) EVP (ASCDP/CD-MOF-1 = 1/2); (**l**) EVP (ASCDP/CD-MOF-1 = 1/4). ●: γ-CD original, ■: CD-MOF-1 original (α type), □: CD-MOF-1 original (β type), ▲: ASCP original, △: ASCDP original.

PXRD measurements were carried out to investigate the crystal changes in the ASCP(ASCDP)/CD-MOF-1 complex. The characteristic diffraction peaks of γ-CD were observed at 2θ = 8.98 and 17.6° (Figure 2a). In the case of CD-MOF-1, the characteristic diffraction peak at 2θ = 7.34° was observed (Figure 2b), while the γ-CD peak was not observed. In EVP (CD-MOF-1), diffraction peaks at 2θ = 6.9, 13.2, and 16.5° were observed, which were distinctive from those of CD-MOF-1 (Figure 2c). Patyk-Kaźmierczak et al. reported that there are two types of CD-MOF-1, α- and β-, and that the difference in water content causes structural changes [10]. Therefore, it was confirmed that CD-MOF-1 underwent a phase transition from the α-form to the β-form following solvent removal. The characteristic diffraction peaks of ASCP were observed at 2θ = 5.6 and 15.5° (Figure 2d). Then, in PM (ASCP/CD-MOF-1 = 1/1), the peaks derived from ASCP and CD-MOF-1 were identified (Figure 2e). However, in the case of EVP (ASCP/CD-MOF-1), the characteristic peaks of ASCP disappeared in both molar ratios (1/1 and 1/2), and similar peaks to those of EVP (CD-MOF-1) were observed (Figure 2f,g).

Subsequently, the characteristic ASCDP-derived diffraction peaks were observed at 2θ = 6.0 and 20.7° (Figure 2h). In the PM (ASCDP/CD-MOF-1 = 1/2), the peaks derived from ASCDP and CD-MOF-1 were identified (Figure 2i). In the case of EVP (ASCDP/CD-MOF-1), the characteristic peaks of ASCDP disappeared and similar peaks to those of EVP (CD-MOF-1) were observed (Figure 2j–l). In other words, new diffraction peaks were observed in EVP (ASCDP/CD-MOF-1 = 1/2) and EVP (ASCDP/CD-MOF-1 = 1/4) as well. It can be presumed that the number of molecules of ASCDP that can form inclusion complex with CD-MOF-1 is 4 for every molecule of ASCDP. Lv et al. reported that CD-MOF-1 showed characteristic crystal diffraction peaks around 2θ = 5.7, 7.0, and 16.7° upon neutralization [27,28]. In other words, the diffraction pattern of EVP (ASCP/CD-MOF-1) is similar to that of the complex formation, suggesting that ASCP/CD-MOF-1 and ASCDP/CD-MOF-1 form a complex upon solvent removal, and the structure of CD-MOF-1 is similar to the β-type.

### 3.2. Evaluation of Thermal Behavior

**Figure 3:**
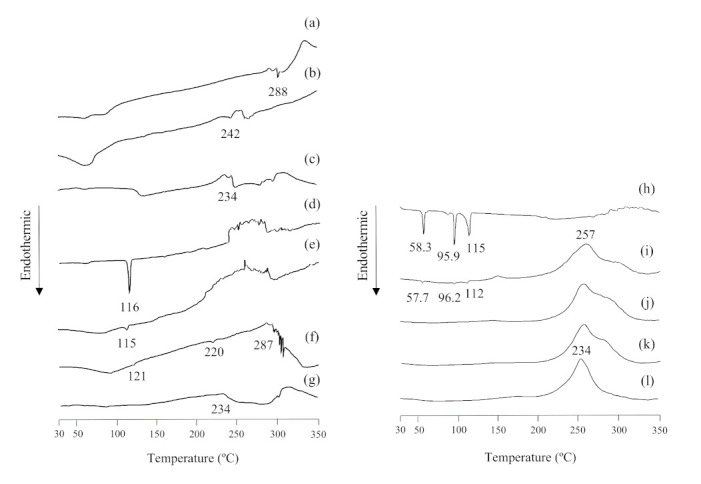
DSC curves of: (**a**) γ-CD; (**b**) CD-MOF-1; (**c**) EVP (CD-MOF-1); (**d**) ASCP intact; (**e**) PM (ASCP/CD-MOF-1 = 1/1); (**f**) EVP (ASCP/CD-MOF-1 = 1/1); (**g**) EVP (ASCP/CD-MOF-1 = 1/2); (**h**) ASCDP intact; (**i**) PM (ASDCP/CD-MOF-1 = 1/1); (**j**) EVP (ASCDP/CD-MOF-1 = 1/1); (**k**) EVP (ASCDP/CD-MOF-1 = 1/2); (**l**) EVP (ASCDP/CD-MOF-1 = 1/4).

Results of the PXRD measurements inferred the formation of ASCP/CD-MOF-1 and ASCDP/CD-MOF-1 complexes. Therefore, DSC measurements were carried out to confirm the thermal behavior. γ-CD showed a peak at 50–100 °C due to dehydration of adsorbed water and a peak at 288 °C due to decomposition (Figure 3a). On the other hand, CD-MOF-1 showed a peak at 50–100 °C due to dehydration of adsorbed water and a peak at 242 °C due to degradation (Figure 3b). In the case of EVP (CD-MOF-1), a degradation point similar to that of CD-MOF-1 was observed at around 234 °C, and an endothermic peak due to dehydration of hydrated water from CD-MOF-1-β was observed at around 132 °C (Figure 3c). Therefore, it was assumed that the properties of CD-MOF-1 and EVP (CD-MOF-1) were different. In ASCP, an endothermic peak derived from the melting point of ASCP was observed at around 116 °C (Figure 3d). For PM (ASCP/CD-MOF-1 = 1/1), endothermic peaks derived from CD-MOF-1 and ASCP were observed, respectively (Figure 3e). In EVP (ASCP/CD-MOF-1 = 1/1), a characteristic peak derived from ASCP was observed at around 121 °C, and a decomposition point was observed at around 287 °C (Figure 3f). However, in EVP (ASCP/CD-MOF-1 = 1/2), the characteristic endothermic peak of ASCP disappeared, and the decomposition point was observed at around 234 °C (Figure 3g). In EVP (ASCP/CD-MOF-1 = 2/1), the characteristic peak of ASCP was observed at around 112 °C, and the decomposition point was observed at around 280 °C.

In terms of endothermic peaks, the melting point of ASCDP was observed at around 58.3, 95.9, and 115 °C (Figure 3h). For PM (ASCDP/CD-MOF-1 = 1/4), endothermic peaks derived from CD-MOF-1 and ASCDP were observed, and a decomposition point was observed at around 257 °C (Figure 3i). In EVP (ASCDP/CD-MOF-1 = 1/2) and EVP (ASCDP/CD-MOF-1 = 1/4), the endothermic peaks characteristic of CD-MOF-1 and ASCDP disappeared, and a decomposition point was identified at around 234 °C (Figure 3k,l). Lv et al. reported that when CD-MOF-1 forms a complex, the endothermic peak derived from the melting point of the guest molecule disappears [26]. Therefore, it was inferred that ASCP/CD-MOF-1 and ASCDP/CD-MOF-1 formed a complex at a molar ratio of (1/2) and (1/4), respectively.

### 3.3. Evaluation of Intermolecular Interaction

**Figure 4:**
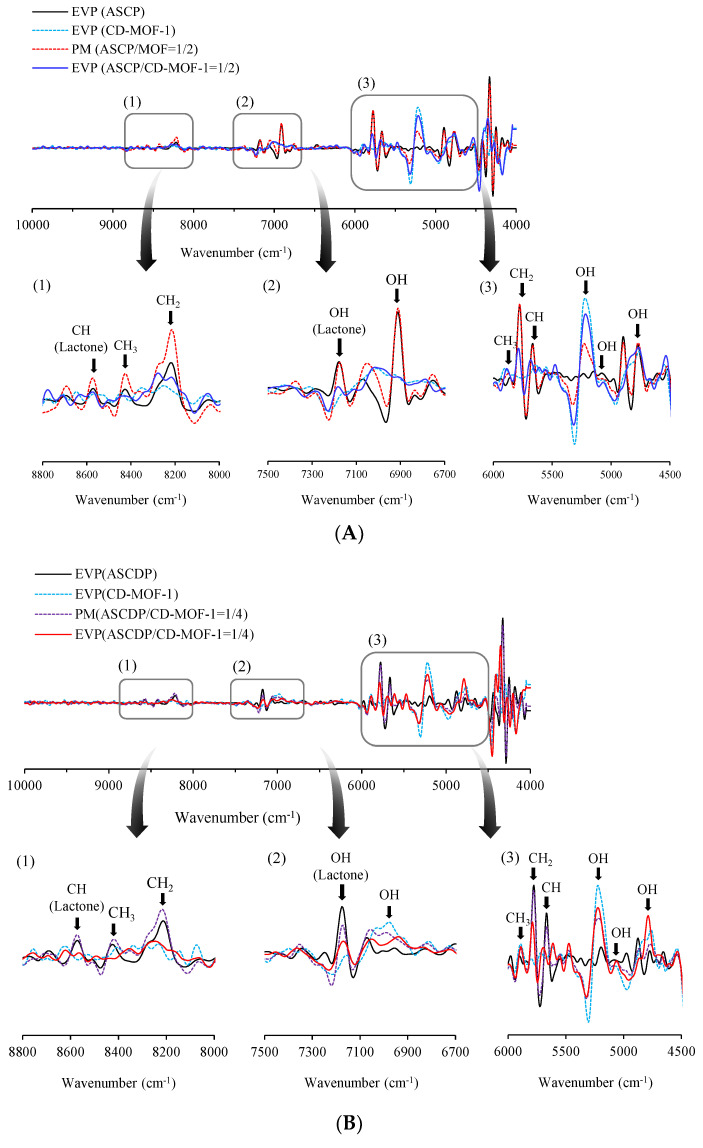
(**A**) Second differentiation NIR absorption spectra of ASCP/CD-MOF-1 systems: (**1**) 8800–8000 cm^−1^; (**2**) 7500–6700 cm^−1^; (**3**) 6000–4500 cm^−1^. (**B**) Second differentiation NIR absorption spectra of ASCDP/CD-MOF-1 systems: (**1**) 8800–8000 cm^−1^; (**2**) 7500–6700 cm^−1^; (**3**) 6000–4500 cm^−1^.

From the results of the PXRD and DSC measurements, the complex formation was estimated at the molar ratios of ASCP/CD-MOF-1 = 1/2 and ASCDP/CD-MOF-1 = 1/4. Therefore, NIR measurements were performed to confirm the intermolecular interaction between ASCP (ASCDP) and CD-MOF-1. In contrast to infrared absorption spectroscopy (IR), NIR is less sensitive to water molecules and is therefore the most suitable method to confirm the intermolecular interaction of CD-MOF-1 with high water content. First, to confirm the effect of solvent removal on the CH and OH groups of CD-MOF-1, a comparison between CD-MOF-1 and EVP (CD-MOF-1) was carried out (Figure 4A). In EVP (CD-MOF-1), the free water origin (–OH group) of CD-MOF-1 (5240 cm^−1^) was shifted to 5212 cm^−1^ (Figure 4(A3)). Furthermore, the peak of the -OH group (5048 cm^−1^) derived from CD-MOF-1 was shifted to 5064 cm^−1^ in EVP (CD-MOF-1), and the peak was confirmed to be sharper. The OH peak (7100 cm^−1^) derived from CD-MOF-1 was shifted to 7040 cm^−1^ in EVP (CD-MOF-1). These results confirmed that EVP (CD-MOF-1) and CD-MOF-1 unprocessed showed different molecular behaviors under different moisture conditions. We then compared these results with those of ASCP alone and EVP (ASCP/CD-MOF-1 = 1/2). The peaks of CH group derived from ASCP were observed around 5620, 5720, and 8336 cm^−1^ (Figure 4(A3)). In EVP (ASCP/CD-MOF-1 = 1/2), the peaks of the CH group from ASCP were shifted to 5612 cm^−1^, 5704 cm^−1^, and 8316 cm^−1^. No shift was observed for the CH and OH peaks of the ASCP-derived lactone ring, suggesting no intermolecular interaction with the lactone ring (Figure 4(A2)). The OH peak of the free water of CD-MOF-1 (5240 cm^−1^) shifted to a higher wavenumber, and the OH peak of CD-MOF-1 (5048 cm^−1^) shifted to lower wavenumber and broadened, suggesting that the hydrogen bonding of the OH group of CD-MOF-1 changed due to the decrease in the amount of free water. This suggested a change in the hydrogen bonding of the OH group from CD-MOF-1 due to the decrease in free water. Furthermore, in EVP (ASCP/CD-MOF-1 = 1/2), the CH peak derived from CD-MOF-1 (8320 cm^−1^) was shifted to 8340 cm^−1^ in EVP (ASCP/CD-MOF-1 = 1/2) (Figure 4(A1)). These results confirmed that EVP (ASCP/CD-MOF-1 = 1/2) and CD-MOF-1 interacted differently in different water environments. Similar to our previous study, it was observed that the shift and broadening of CH groups from CoQ10 and CD-MOF-1 occurred upon solvent removal due to changes in the water environment associated with changes in the hydrogen bonding of OH groups from CD-MOF-1 [26]. Therefore, the formation of the ASCP/CD-MOF complex was suggested by intermolecular interactions in the hydrogen bonds between the CH group of the side chain of ASCP and the OH group of CD-MOF-1.

Next, a comparison was made between ASCDP alone and EVP (ASCDP/CD-MOF-1 = 1/2). The peak of the -CH group from ASCDP was observed at around 8428 cm^−1^ (Figure 4(B1)). In EVP (ASCDP/CD-MOF-1 = 1/2), the peak of CH group from ASCDP was shifted to 8432 cm^−1^. The CH peak group of the lactone ring from ASCDP was observed at 8572 cm^−1^, and the OH peak of the lactone ring was observed at around 7172 cm^−1^(Figure 4(B2)). In EVP (ASCDP/CD-MOF-1 = 1/2), the CH peak group of lactone ring from ASCDP and the OH peak of the lactone ring shifted to 8576 cm^−1^ and 7168 cm^−1^, respectively. The OH peak of free water of EVP (CD-MOF-1) (5212 cm^−1^) was shifted to a higher wavenumber, and the OH peak of CD-MOF-1 (5048 cm^−1^) also was shifted to higher wavenumber, and sharpened. These results suggested that the decrease in free water led to a change in the hydrogen bonding of the OH group derived from CD-MOF-1. Furthermore, the peak of CH group from CD-MOF-1 (8292 cm^−1^) was shifted to 8260 cm^−1^ in EVP (ASCDP/CD-MOF-1 = 1/2) and broadened in EVP (ASCDP/CD-MOF-1 = 1/2).

The peak of the CH group from ASCDP was observed at around 8428 cm^−1^ (Figure 4(B1)). The CH peak of the lactone ring from ASCDP was observed at 8572 cm^−1^, and the OH peak of the lactone ring was observed at around 7172 cm^−1^ (Figure 4(B2)). In EVP (ASCDP/CD-MOF-1 = 1/4), the CH peak of the lactone ring from ASCDP was shifted to 8564 cm^−1^, and that of the OH group of the lactone ring to 7176 cm^−1^. The OH peak of free water in EVP (CD-MOF-1) (5212 cm^−1^) was shifted to a higher wavenumber, and the OH peak of CD-MOF-1 (5048 cm^−1^) also was shifted to higher wavenumber, and sharpened. (Figure 4(B3)). In comparison to EVP (ASCDP/CD-MOF-1 = 1/2), the peak of CH group in EVP (ASCDP/CD-MOF-1 = 1/4), originating from CD-MOF-1 (8292 cm^−1^), was shifted to 8248 cm^−1^ and broadened, indicating that EVP (ASCDP/CD-MOF-1 = 1/2, 1/4) and CD-MOF-1 interacted differently in different water environments. In a previous study, it was observed that the CH groups of CoQ10 and CD-MOF-1 shifted and broadened with the change in water environment due to the change in hydrogen bonding of the OH group of CD-MOF-1 upon solvent removal [26]. Therefore, the formation of a complex between the CH group of ASCDP and the OH group of CD-MOF-1 was suggested by the intermolecular interaction due to hydrogen bonding. This suggested that the interaction with CD-MOF-1 occurred via the palmitoyl groups at positions 2 and 6 in ASCDP.

### 3.4. Evaluation of Morphological Properties

**Figure 5:**
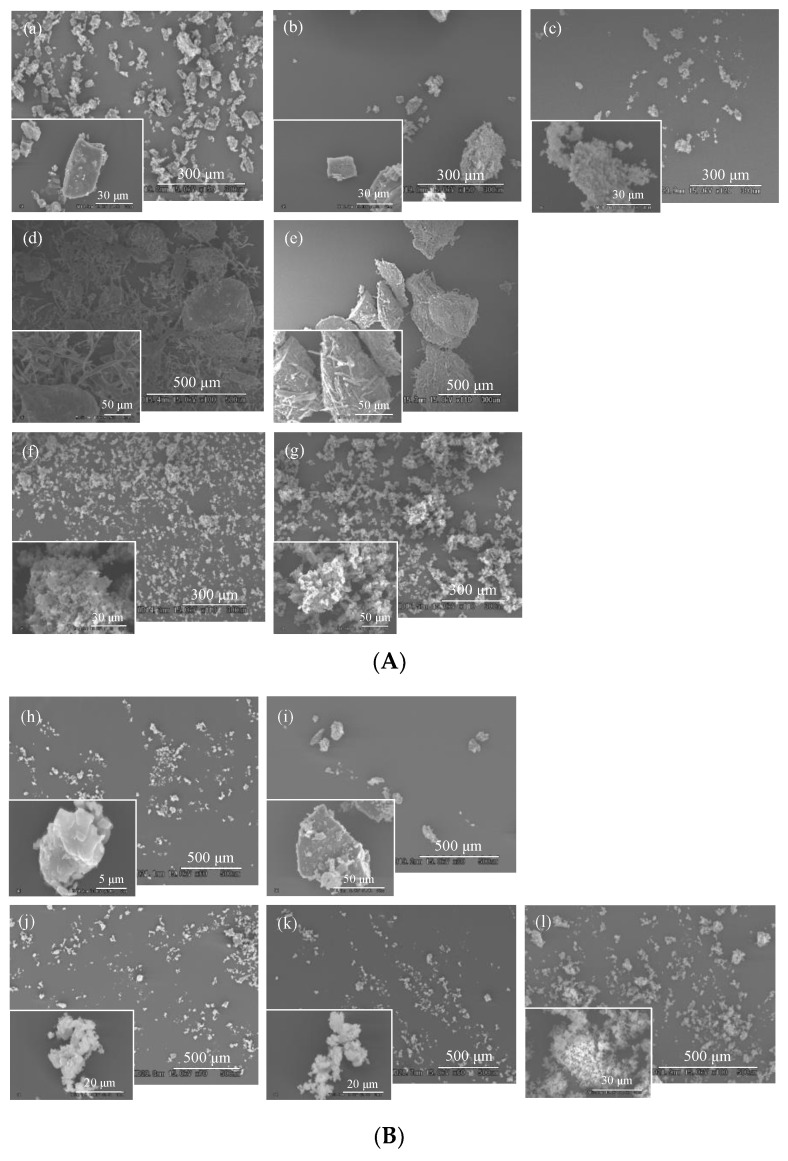
(**A**) SEM micrographs of: (**a**) γ-CD; (**b**) CD-MOF-1; (**c**) EVP (CD-MOF-1); (**d**) ASCP intact; (**e**) PM (ASCP/CD-MOF-1 = 1/2); (**f**) EVP (ASCP/CD-MOF-1 = 1/1); (**g**) EVP (ASCP/CD-MOF-1 = 1/2). (**B**) SEM micrographs of: (**h**) ASCDP intact; (**i**) PM (ASCDP/CD-MOF-1 = 1/4); (**j**) EVP (ASCDP/CD-MOF-1 = 1/1); (**k**) EVP (ASCDP/CD-MOF-1 = 1/2); (**l**) EVP (ASCDP/CD-MOF-1 = 1/4).

The formation of complexes was suggested at ASCP/CD-MOF-1 = 1/2 and ASCDP/CD-MOF-1 = 1/4. Microscopic observations in SEM confirmed the particle size and morphological changes in ASCP/CD-MOF-1 and ASCDP/CD-MOF-1. The surface of γ-CD particles was smooth and elongated, with a diameter of about 60 μm (Figure 5a). In the case of CD-MOF-1, the surface of the particles was smooth, and the size of the particles was about 30 μm (Figure 5b). Li et al. reported that CD-MOF-1 exhibited a cubic packing structure [27]. EVP (CD-MOF-1) was found to have an aggregation of cubic crystals of less than 2 μm on its surface (Figure 5c). In ASCP, the surface of the particles was found to be coarse and agglomerated with needlelike crystals (Figure 5d). In PM (ASCP/CD-MOF-1 = 1/2), ASCP was found to be agglomerated around CD-MOF-1 crystals (Figure 5e). Similarly, in EVP (ASCP/CD-MOF-1 = 1/1), ASCP was found to aggregate around the CD-MOF-1 crystals (Figure 5f). However, in EVP (ASCP/CD-MOF-1 = 1/2), cubic crystals of less than 2 µm in size were found aggregated on the surface (Figure 5g). Lv et al. reported that neutralization of CD-MOF-1 disintegrated a portion of CD-MOF-1 into fine particles [28]. Furthermore, in EVP (ASCP/CD-MOF-1 = 1/2), no particles of ASCP were observed, suggesting that the morphological changes of these particles originate from the complex formation. In the case of ASCDP, the particle surface was rough, and an elongated, rounded shape with a particle size of about 10 μm was observed (Figure 5h). In PM (ASCDP/CD-MOF-1 = 1/4), ASCDP was found to be aggregated around CD-MOF-1 crystals (Figure 5i). Similarly, in EVP (ASCDP/CD-MOF-1 = 1/1) and EVP (ASCDP/CD-MOF-1 = 1/2), ASCDP was found to be aggregated around CD-MOF-1 crystals (Figure 5j,k)). However, in EVP (ASCDP/CD-MOF-1 = 1/4), cubic crystals of less than 2 µm in size were found aggregated on the surface (Figure 5l). In EVP (ASCDP/CD-MOF-1 = 1/4), no ASCDP particles were observed, suggesting that the morphological changes of these particles originated from the complex formation.

### 3.5. Evaluation of Relative Positional Relationship

**Figure 6:**
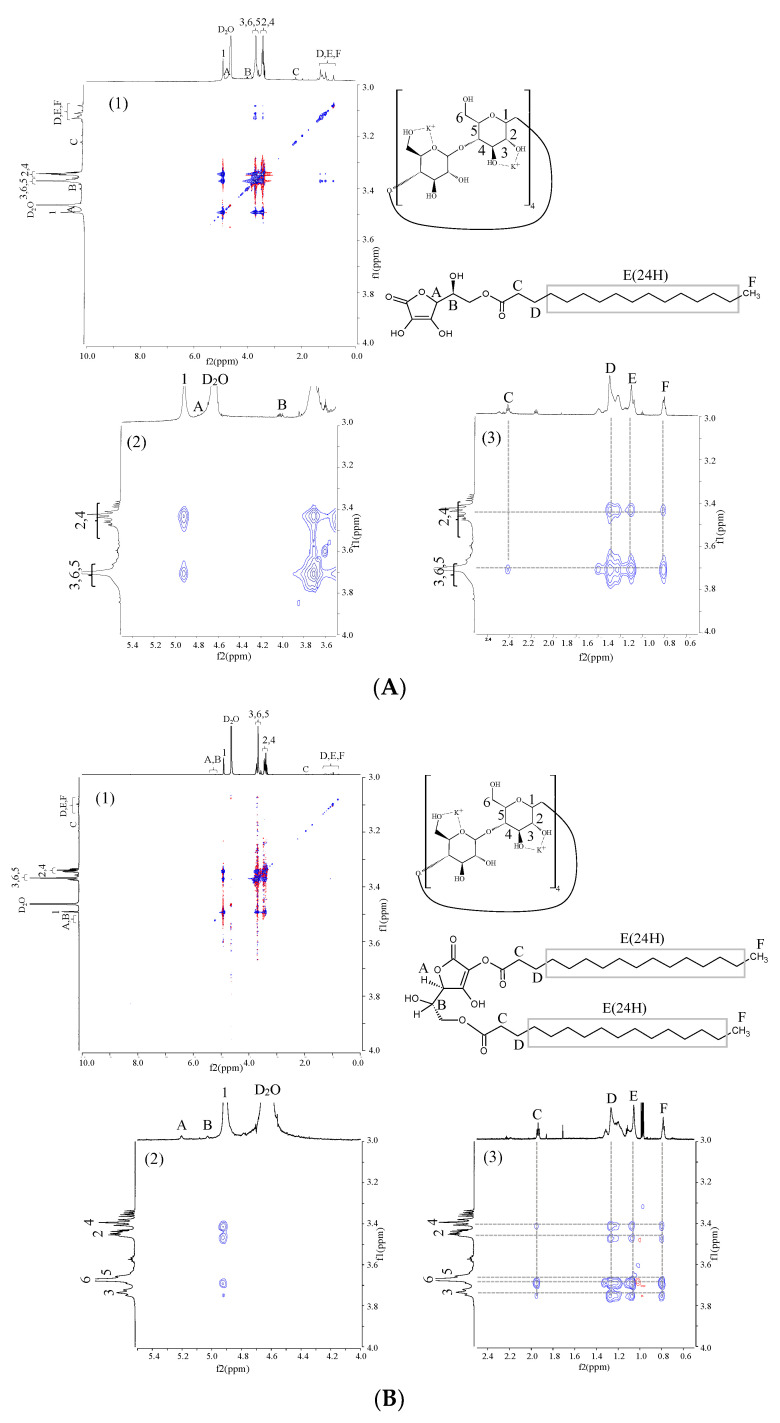
(**A**) ^1^H-^1^H NOESY NMR spectra EVP (ASCP/CD-MOF-1 = 1/2) in D_2_O: (**1**) f1 is 3.0–4.0 ppm, f2 is 0.0–10.0 ppm; (**2**) f1 is 3.0–4.0 ppm, f2 is 3.5–5.5 ppm; (**3**) f1 is 3.0–4.0 ppm, f2 is 0.5–2.5 ppm. (**B**) ^1^H-^1^H NOESY NMR spectra EVP (ASCDP/CD-MOF-1 = 1/4) in D_2_O: (**1**) f1 is 3.0–4.0 ppm, f2 is 0.0–10.0 ppm; (**2**) f1 is 3.0–4.0 ppm, f2 is 4.0–5.5 ppm; (**3**) f1 is 3.0–4.0 ppm, f2 is 0.5–2.5 ppm.

**Scheme 1:**
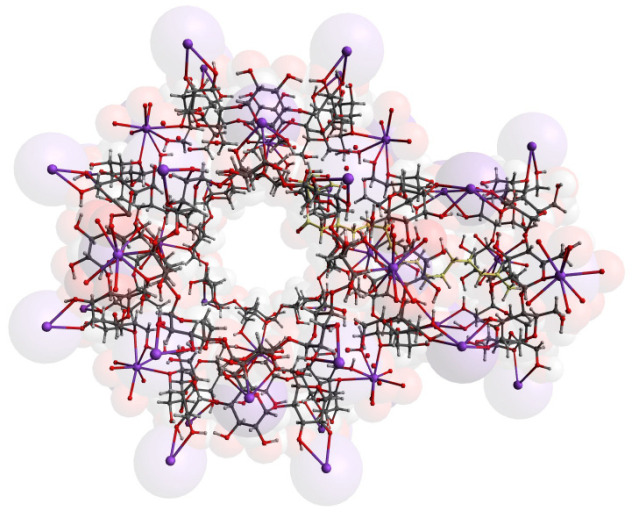
Structural view of ASCP/CD-MOF-1 complex.

In this study, 2D-NOESY NMR measurements were carried out to investigate in detail the intermolecular interactions of EVP (ASCP/CD-MOF-1 = 1/2) and EVP (ASCDP/CD-MOF-1 = 1/4) (Figure 6). The attribution of the ASCP peak was based on the report of Inoue et al. [26]. No cross-peaks with H-A and H-B of ASCP were observed in CD-MOF-1, whereas cross-peaks with H-C (2.0 ppm) of ASCP were observed in H-5 (3.7 ppm), H-3 (3.8 ppm), and H-6 (3.7 ppm) of CD-MOF-1 (Figure 6A). The cross-peaks of H-5 (3.7 ppm), H-3 (3.8 ppm), H-2 (3.5 ppm), H-4 (3.4 ppm), and H-6 (3.7 ppm) of CD-MOF-1 were observed with H-D (1.3 ppm), H-E (1.1 ppm), and H-F (1.0 ppm). This suggested that in EVP (ASCP/CD-MOF-1 = 1/2), the palmitic acid side chain, which is the lipophilic moiety of ASCP, enters the cavity of CD-MOF-1 from the wide rim to the narrow rim and further coordinates from the narrow rim to the wide rim of the neighboring CD-MOF-1. In addition, the lactones of the ASCPs were found to be coordinated from the verify to the narrow rim of the adjacent CD-MOF-1 (Scheme 1).

**Scheme 2:**
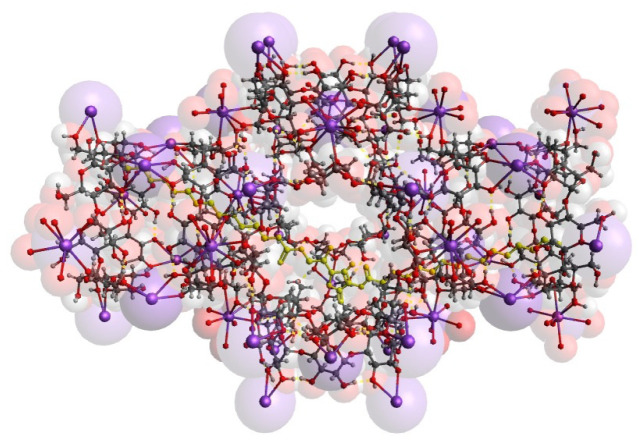
Structural view of ASCDP/CD-MOF-1 complex.

The 2D-NOESY NMR measurements of the ASCDP/CD-MOF system revealed no cross-peaks of CD-MOF-1 with H-A and H-B of ASCDP in EVP (ASCDP/CD-MOF-1 = 1/4) (Figure 6B). Cross-peaks with H-C (2.0 ppm) of ASCDP were observed for H-5,6 (3.7 ppm), H-3 (3.8 ppm), and H-4 (3.4 ppm) of CD-MOF-1. Cross peaks of H-5,6 (3.7 ppm), H-3 (3.8 ppm), H-2 (3.5 ppm), and H-4 (3.4 ppm) of CD-MOF-1 were observed with H-D (1.3 ppm), H-E (1.1 ppm), and H-F (1.0 ppm). In EVP (ASCDP/CD-MOF-1 = 1/4), no cross-peaks of CD-MOF-1 with H-A and H-B of ASCDP were observed. Cross-peaks with H-C (2.0 ppm) of ASCDP were observed for H-5,6 (3.7 ppm), H-3 (3.8 ppm), and H-4 (3.4 ppm), of CD-MOF-1. The cross-peaks of H-5,6 (3.7 ppm), H-3 (3.8 ppm), H-2 (3.5 ppm), and H-4 (3.4 ppm) of CD-MOF-1 were observed with those of H-D (1.3 ppm), H-E (1.1 ppm), and H-F (1.0 ppm). This suggested that in EVP (ASCDP/CD-MOF-1 = 1/4), the palmitic acid side chain, which is the lipophilic moiety of ASCDP, enters from the wide rim to the narrow rim in the cavity of CD-MOF-1, and further coordinates from the narrow rim to the wide rim of the neighboring CD-MOF-1. The lactone ring and the CH group attached to the lactone ring of ASCDP did not show any intermolecular interaction with the interior of CD-MOF-1, suggesting that they were not coordinated within the CD-MOF-1 cavity (Scheme 2). The similarity of the intermolecular interactions of the complexes between EVP (ASCDP/CD-MOF-1 = 1/2) and EVP (ASCDP/CD-MOF-1 = 1/4) demonstrated that the selectivity of CD-MOF-1 for the palmitic acid side chain, which is the lipid-soluble part of ASCDP, was two molecules from the 2-position palmitic acid side chain or two molecules from the 6-position palmitic acid side chain. These results suggest that novel CD-MOF-1 is useful in developing drug carriers for ASCP and ASCDP. Clarification of the inclusion mode with CD-MOF-1 may facilitate future product development by improving the solubility, absorption, and stability of various lipophilic compounds with side chains.

## 4. Conclusions

In the present study, PXRD and DSC results suggested that the complexes were formed at molar ratios of (1/2) for ASCP/CD-MOF-1 and (1/4) for ASCDP/CD-MOF-1. Intermolecular interactions were observed in the hydrogen bonds between the CH group of the side chain of ASCP and the OH group of CD-MOF-1. Therefore, the formation of complexes of ASCP/CD-MOF-1 and ASCDP/CD-MOF-1 was initiated by solvent removal. In 2D-NOESY NMR measurements, CD-MOF-1 was found to be in an inclusion complex with the side chain, the lipophilic moiety of ascorbic acid palmitate. In the case of ASCDP/CD-MOF-1, two molecules of CD-MOF-1 from one side of the palmitic acid side chain were found to be incorporated selectively in the palmitic acid side chain. The present results suggested that CD-MOF-1 was selective for inclusion with either of the side chain when the liposoluble drug had multiple side chains. MOF-1 successfully produced novel drug carrier systems for L-ascorbyl 6-palmitate (ASCP) and L-Ascorbyl 2,6-palmitate (ASCDP).

## Abbreviations

MOFs	Metal–organic frameworks
γ-CD	γ-Cyclodextrin
CD-MOF-1	Cyclodextrin-based metal–organic frameworks-1
ROS	Reactive oxygen species
ASC	L(+)-ascorbic Acid
ASCP	L-ascorbyl 6-palmitate
ASCDP	L-ascorbyl 2, 6-palmitate
CoQ10	Coenzyme Q10
FaSSIF	Fasted state simulated intestinal fluid
PM	Physical mixture
EVP	Evaporated sample
PXRD	Powder X-ray diffraction
DSC	Differential scanning calorimetry
NIR	Near-infrared absorption spectroscopy
SEM	Scanning electron microscopy
NOESY	Nuclear Overhauser effect spectroscopy
IR	Infrared absorption spectroscopy
